# Neuroprotective Effects of Food‐Derived Cyanidin Glycosides in an Alzheimer's Disease‐Like Zebrafish Model Through Modulation of PI3K/Akt/GSK3β Signaling

**DOI:** 10.1002/fsn3.72486

**Published:** 2026-10-15

**Authors:** Yun Wu, Fangfang Tie, Yidan Gao, Jinze Li, Ruinan Wang, Na Hu, Qi Dong, Honglun Wang

**Affiliations:** ^1^ Qinghai Provincial Key Laboratory of Tibetan Medicine Research, Northwest Institute of Plateau Biology Chinese Academy of Sciences Xining China; ^2^ University of Chinese Academy of Sciences Beijing China

**Keywords:** Alzheimer's disease, cyanidin glycosides, neuroprotection, PI3K/Akt/GSK3β signaling pathway, zebrafish

## Abstract

Alzheimer's disease (AD) is a progressive neurodegenerative disorder lacking effective and safe interventions. Food‐derived anthocyanins have gained increasing attention due to their neuroprotective potential; however, the specific mechanisms of individual cyanidin glycosides remain insufficiently understood. Notably, the comparative efficacy and mechanistic differences among structurally distinct cyanidin glycosides have not been systematically evaluated in vivo. In the present study, three cyanidin glycosides, cyanidin‐3‐*O*‐rutinoside (C3R), cyanidin‐3‐*O*‐sophoroside (C3S), and cyanidin‐3‐*O*‐xyloside (C3X), were evaluated using an aluminum chloride (AlCl_3_)‐induced zebrafish model of AD‐like pathology. The results showed that these compounds improved cognitive performance and anxiety‐like behavior, while attenuating oxidative stress and neuroinflammation and restoring cholinergic function, as evidenced by biochemical and immunofluorescence analyses. In addition, neuronal damage and Tau phosphorylation at Ser396 were markedly alleviated. Among the tested compounds, C3R consistently exhibited the most pronounced effects across multiple indicators. Mechanistically, the three cyanidin glycosides modulated PI3K/Akt/GSK3β signaling, as reflected by overall increases in p‐PI3K (Tyr458)/PI3K and p‐Akt (Ser473)/Akt and decreases in p‐GSK3β (Tyr216)/GSK3β and p‐Tau (Ser396)/Tau. LY294002 attenuated the pathway‐associated protective effects, supporting the involvement of PI3K/Akt signaling. Collectively, these findings demonstrate neuroprotective effects associated with modulation of PI3K/Akt/GSK3β signaling, with C3R showing the strongest overall efficacy.

## Graphical abstract

Three food‐derived cyanidin glycosides (C3R, C3S, and C3X) showed neuroprotective effects in an AlCl_3_‐induced Alzheimer's disease‐like zebrafish model, alleviating behavioral deficits, oxidative stress, neuroinflammation, and neuronal damage. C3R exhibited the strongest overall efficacy, with the protective effects associated with modulation of the PI3K/Akt/GSK3β signaling pathway.

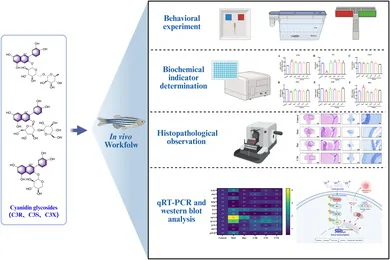

**Abbreviations**
ACh: acetylcholine
AChE: acetylcholinesterase
AD: Alzheimer's disease
Akt: protein kinase B
C3G: cyanidin‐3‐*O*‐glucoside
C3R: cyanidin‐3‐*O*‐rutinoside
C3S: cyanidin‐3‐*O*‐sophoroside
C3X: cyanidin‐3‐*O*‐xyloside
ChAT: choline acetyltransferase
Dpz: donepezil
FO: familiar object
GAPDH: glyceraldehyde‐3‐phosphate dehydrogenase
GSH: glutathione
GSK3β: glycogen synthase kinase‐3β
MDA: malondialdehyde
NO: novel object
NOR: novel object recognition test
NTT: novel tank test
PI3K: phosphatidylinositol 3‐kinase
SOD: superoxide dismutase

## 1 Introduction

Alzheimer's disease (AD) is a progressive neurodegenerative disorder characterized by an insidious onset and gradual cognitive decline (Klonarakis et al. 2022; Mahaman et al. 2022). Its clinical manifestations include memory impairment, aphasia, apraxia, agnosia, visuospatial dysfunction, and deficits in executive function (Pan et al. 2022), often accompanied by behavioral and personality changes (Porsteinsson et al. 2021). As life expectancy increases, the incidence and prevalence of AD continue to rise worldwide (Fu and Ip 2023), and the disease has become a major cause of mortality among older adults (Zhang et al. 2021). According to Alzheimer's Disease International (ADI), a new case of AD dementia occurs approximately every 3 s worldwide (Twiss et al. 2025) and the number of people living with dementia is projected to exceed 152 million by 2050 (Scheltens et al. 2021; Vitali et al. 2021). This rapidly growing burden places considerable pressure on patients, families, and healthcare systems, making AD one of the most pressing challenges in geriatric medicine (Rafe 2024; Şimşek and Uçar 2024).

Natural products continue to provide an important source of bioactive compounds for drug discovery and development (Singh et al. 2025). Several acetylcholinesterase inhibitors (AChEIs) used in AD treatment, such as galantamine and huperzine A, are derived from plants (Gajendra et al. 2024), illustrating the potential of naturally occurring compounds as sources of neuroprotective agents (Andrade et al. 2023). Among food‐derived phytochemicals, cyanidin is a widely distributed anthocyanidin that occurs predominantly in glycosylated forms in dark‐colored plant foods, including black rice, black beans, and purple sweet potatoes (Galvano et al. 2004; Wang et al. 2025). Cyanidin glycosides are commonly consumed through fruits, vegetables, legumes, and red wine, supporting their relevance as dietary bioactive compounds (Rudrapal et al. 2022; Xi et al. 2024). Accumulating evidence indicates that cyanidin and its derivatives exert protective effects in various neurological disorders, including AD, Parkinson's disease, and glioblastoma (X. N. Li, Fan, et al. 2025; L. Liu, Ma, et al. 2025; Zhou et al. 2022). Cyanidin‐3‐*O*‐glucoside (C3G), one of the most abundant and extensively studied cyanidin glycosides, has been reported to cross the blood–brain barrier following oral administration and to accumulate in brain tissue, where it exerts neuroprotective effects (Baek et al. 2023; J. Li, Chen, et al. 2025).

Our previous screening evaluated five cyanidin derivatives, including cyanidin‐3‐*O*‐(*trans*‐*p*‐coumaroyl)‐diglycoside (C3GG), cyanidin‐3‐*O*‐rutinoside (C3R), cyanidin‐3‐*O*‐arabinoside (C3A), cyanidin‐3‐*O*‐sophoroside (C3S), and cyanidin‐3‐*O*‐xyloside (C3X) (Wu et al. 2025). Among these compounds, C3R, C3S, and C3X showed the most promising activities and were therefore selected for further investigation. These compounds contain distinct rutinoside, sophoroside, and xyloside substitutions, respectively. However, most previous studies have focused on individual cyanidin compounds, and direct in vivo evaluations of structurally distinct cyanidin glycosides remain limited. In particular, whether different glycosylation patterns influence neuroprotective activity and the regulation of cholinergic function and PI3K/Akt/GSK3β signaling has not been systematically investigated.

The PI3K/Akt signaling pathway plays a crucial role in regulating neuronal survival, differentiation, and apoptosis. Activation of PI3K promotes Akt phosphorylation, thereby supporting neuronal survival and limiting apoptosis (T. Liu, Li, et al. 2025). Activated Akt further regulates downstream targets, including GSK3β, and impaired PI3K/Akt signaling in AD‐related pathology may result in insufficient inhibition of GSK3β (Kitagishi et al. 2014). As a downstream serine/threonine kinase, overactivated GSK3β promotes Tau hyperphosphorylation and neuronal apoptosis (Chauhan et al. 2022). Therefore, the PI3K/Akt/GSK3β pathway is considered a potential therapeutic target for AD. Several natural compounds, including salidroside (Wang et al. 2023), arctigenin (Qi et al. 2017), and asiatic acid (Cheng et al. 2018), have been reported to exert neuroprotective effects in association with modulation of PI3K/Akt/GSK3β signaling. These observations provide a biological rationale for investigating whether the neuroprotective effects of cyanidin glycosides are associated with this pathway and whether their different sugar moieties contribute to distinct pathway responses.

Zebrafish ( *Danio rerio* ) are widely used as a complementary vertebrate model that bridges the gap between in vitro experiments and mammalian studies (Pozo‐Morales et al. 2023). Zebrafish behavioral assays permit robust and quantifiable assessment of sensorimotor, emotional, learning, and memory functions (Akinrinade et al. 2023), facilitating the rapid screening of bioactive compounds for neurological disorders (Dash and Patnaik 2023). In addition, zebrafish also possess well‐characterized dopaminergic, cholinergic, and noradrenergic neurotransmitter systems (Könemann et al. 2022) and have been extensively employed to investigate neurodegenerative diseases and central nervous system mechanisms (Teng et al. 2022). In particular, AlCl_3_ exposure produces selected AD‐like phenotypes in zebrafish, including cognitive impairment, oxidative stress, and cholinergic dysfunction, making this model suitable for the preliminary in vivo evaluation of food‐derived neuroprotective compounds (Vaja et al. 2025).

In this study, an equal‐dose comparison of C3R, C3S, and C3X was conducted in adult zebrafish with AlCl_3_‐induced AD‐like pathology. Behavioral testing was combined with histological, ultrastructural, biochemical, immunofluorescence, gene‐expression, and protein‐phosphorylation analyses to evaluate cognitive performance, neuronal integrity, cholinergic function, oxidative and inflammatory responses, Tau phosphorylation, and PI3K/Akt/GSK3β signaling. The PI3K inhibitor LY294002 was subsequently employed to examine the functional involvement of this pathway. This study aimed to clarify the differential neuroprotective profiles of structurally distinct cyanidin glycosides and to provide a basis for prioritizing promising food‐derived candidates for further pharmacokinetic and translational evaluation.

## 2 Materials and Methods

### 2.1 Chemicals and Reagents

AlCl_3_ was procured from Sigma‐Aldrich (St. Louis, MO, USA). Donepezil (Dpz) was acquired from Shanghai McLean Biochemical Technology Co. Ltd. (Shanghai, China). The cyanidin glycosides (C3R, C3S, and C3X), food‐derived anthocyanins, were obtained from Shanghai Yuanye Biotechnology Co. Ltd. (Shanghai, China) with HPLC purity ≥ 98%. Phosphate‐buffered saline (PBS) was purchased from Wuhan Pnousai Life Science Co. Ltd. (Wuhan, China). Assay kits for AChE, acetylcholine (ACh), choline acetyltransferase (ChAT), superoxide dismutase (SOD), glutathione (GSH), and malondialdehyde (MDA) were supplied by Shanghai Enzyme‐linked Biotechnology Co. Ltd. and Nanjing Jiancheng Bioengineering Institute. TRIzol reagent and reverse transcription kits were acquired from TaKaRa Biotechnology Co. Ltd. (Dalian, China). The ChamQ Universal SYBR qPCR Master Mix was purchased from Vazyme Biotech Co. Ltd. (Nanjing, China). All primers were synthesized by Shanghai Bioengineering Co. Ltd. (Shanghai, China). The bicinchoninic acid (BCA) protein assay kit and protein lysis buffer were from Beyotime Biotechnology Co. Ltd. (Shanghai, China). LY294002 was obtained from MedChemExpress (cat. no. HY‐10108).

For western blot analysis, primary antibodies against phospho‐PI3K p85 (Tyr458)/p55 (Tyr199) (#17366), PI3K p85 (#4257), phospho‐Akt (Ser473) (#4060), Akt (#4691), phospho‐GSK3β (Tyr216) (#71057), GSK3β (#9315), and GAPDH (#2118) were purchased from Cell Signaling Technology (Danvers, MA, USA). The antibody against phospho‐Tau (Ser396) (ab109390) was purchased from Abcam (Cambridge, UK). Polyclonal antibodies against AChE (17975‐1‐AP) and ChAT (20747‐1‐AP), as well as the HRP‐conjugated goat anti‐rabbit IgG (H + L) secondary antibody (RGAR001), were obtained from Proteintech Group Inc. (Wuhan, China).

### 2.2 Animals and Ethics

Adult wild‐type zebrafish (AB strain) were provided by Hubei Chuangxin Biotechnology Co. Ltd. (Wuhan, China) and maintained in a recirculating aquaculture system (Beijing Aisheng Technology Development Co. Ltd., Beijing, China) under controlled conditions: water temperature 26°C ± 2°C, pH 7.0–8.0, conductivity 500–800 μS/cm, and a 14‐h light/10‐h dark photoperiod. All experimental procedures involving zebrafish were conducted in accordance with the guidelines approved by the Animal Ethics Committee of the Northwest Institute of Plateau Biology, Chinese Academy of Sciences (Approval No. 2025‐37).

### 2.3 Establishment of the AD‐Like Zebrafish Model and Compound Administration

An AlCl_3_‐induced AD‐like zebrafish model, based on previously reported protocols (Kaur et al. 2022; Nie et al. 2022), was employed to induce AD‐like pathology. Adult zebrafish were used instead of larvae to better reflect neurodegenerative changes in mature organisms. The zebrafish were exposed to AlCl_3_ (100 μg/L) in water for 4 consecutive days, overlapping with the 5‐day gavage regimen described below. On Day 4, all AlCl_3_‐exposed groups additionally received an intraperitoneal (i.p.) injection of AlCl_3_ solution (4.2 mg/mL, 10 μL/fish) to reinforce model induction.

Before the main efficacy experiment, a preliminary dose–response screening was conducted using Dpz, C3R, C3S, and C3X at 0.5, 1, and 2 mg/kg. Brain AChE, ChAT, SOD, GSH, and MDA were assessed as indicators of cholinergic function and oxidative stress (Figure S1). Based on the overall response profiles of these markers, 2 mg/kg, the highest dose evaluated in the preliminary screening, was selected as the common dose for the subsequent equal‐dose comparison of C3R, C3S, and C3X. This dose was chosen to standardize the comparison among compounds rather than to define the compound‐specific maximum effective dose.

Zebrafish were randomly assigned to six groups (*n* = 15 per group): Control, Model, Positive control (Dpz, 2 mg/kg), C3R (2 mg/kg), C3S (2 mg/kg), and C3X (2 mg/kg). Dpz, C3R, C3S, and C3X were administered once daily by intragastric gavage (10 μL/fish) for 5 consecutive days, beginning on the first day of AlCl_3_ exposure. The Control and Model groups received an equivalent volume of saline according to the same gavage schedule. The final gavage was administered on Day 5 before endpoint assessments and tissue collection. The experimental grouping, model induction and treatment, behavioral assessment, brain tissue collection, and endpoint analyses are summarized in Figure 1.

**FIGURE 1:**
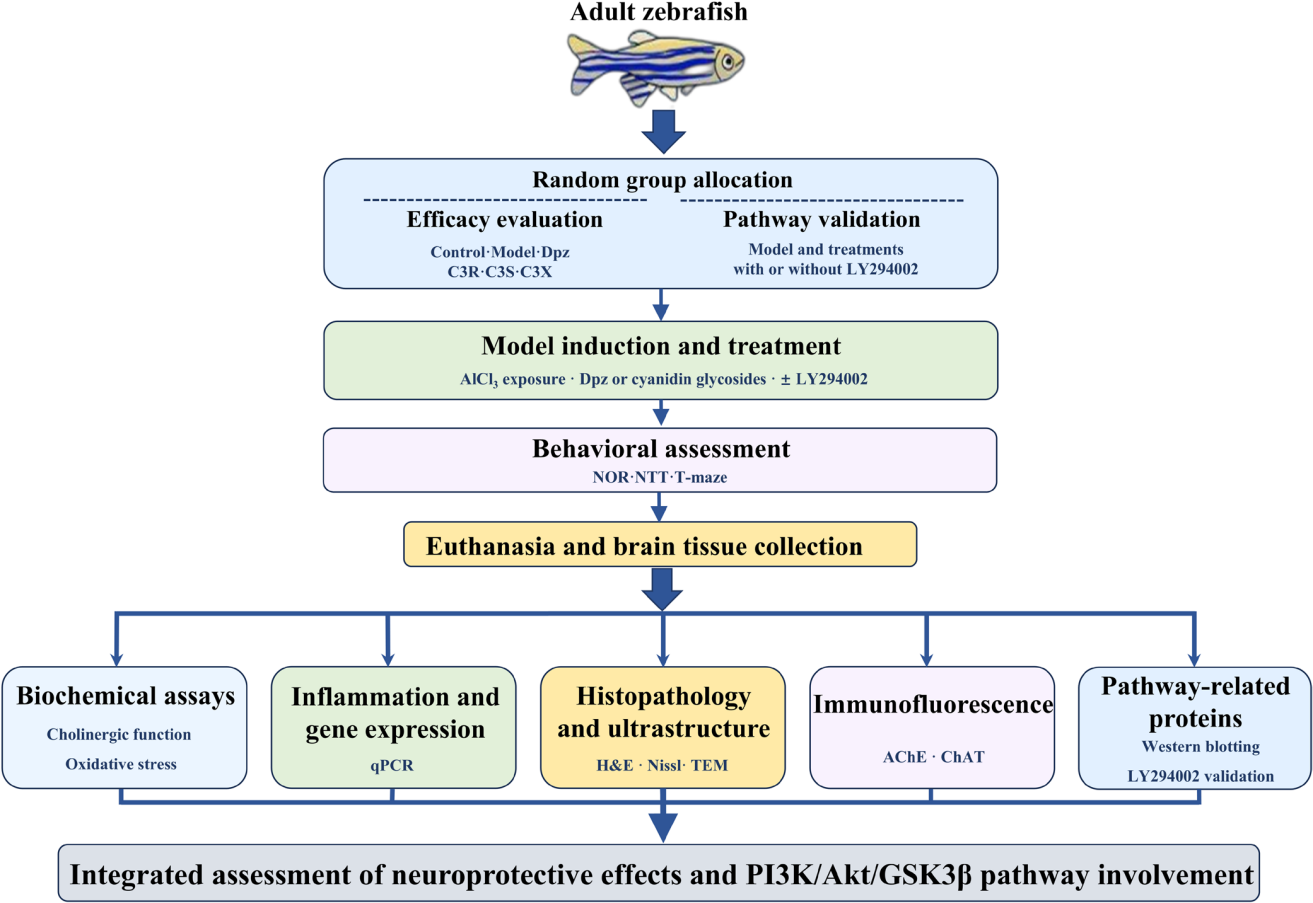
Schematic overview of the adult zebrafish experimental workflow.

To determine whether the PI3K/Akt/GSK3β pathway contributed to the effects of the cyanidin glycosides, a separate pathway‐validation experiment was conducted using only AlCl_3_‐exposed zebrafish. Zebrafish were randomly assigned to nine groups (*n* = 6 per group): Model, Model + C3R, Model + C3R + LY294002, Model + C3S, Model + C3S + LY294002, Model + C3X, Model + C3X + LY294002, Model + LY294002, and Model + Dpz. C3R, C3S, C3X, and Dpz were each administered at 2 mg/kg. In the four LY294002‐containing groups, LY294002 (2 mg/kg, i.p.) was administered 2 h before daily gavage. The Model + LY294002 group received LY294002 without Dpz or any cyanidin glycoside and therefore served as the inhibitor‐only group under the model condition. The model‐induction and gavage procedures were otherwise identical to those used in the efficacy experiment. After the final treatment, brain tissues were collected for Western blotting.

### 2.4 Behavioral Analysis

To evaluate AD‐related behavioral alterations, an automated video‐tracking system was used. Individual zebrafish were placed in testing tanks, and their swimming activity was recorded with a monochrome camera. Behavioral data were analyzed using VisuTrack AI software (Shanghai Xinruan Information Technology Co. Ltd., Shanghai, China) to quantify parameters such as total distance traveled and average velocity.

#### 2.4.1 Novel Object Recognition Test (NOR)

The NOR test is commonly used to assess memory function and anxiety‐like behavior in zebrafish (May et al. 2016). The test was conducted in a square acrylic tank (30 × 30 × 30 cm) filled with water to a depth of 6 cm. Zebrafish were acclimated to the apparatus without objects for 30 min per day over two consecutive days. On the third day, each zebrafish was allowed to explore two identical red objects for 10 min (training phase). After 1 h, one of the familiar objects was replaced with a novel blue object, and exploration time around each object was recorded for 10 min.

#### 2.4.2 Novel Tank Test (NTT)

The NTT was used to evaluate vertical exploration and anxiety‐like behavior in zebrafish (Kysil et al. 2017). The test was conducted in a tank (2.5 L) filled with water to a depth of 10 cm. The tank was divided into top and bottom zones using a virtual horizontal line. Each zebrafish was individually placed in the tank. After a 1 min habituation period, behavior was recorded for 5 min. The following parameters were recorded: latency to enter the top zone (s), total distance traveled (mm), and average velocity (mm/s).

#### 2.4.3 T‐Maze Test

The color‐biased T‐maze test was performed according to the method described by Singsai et al. (2024). The apparatus consisted of a transparent T‐shaped maze filled with water to a depth of 8 cm, including one long arm and two short arms (red and green). Zebrafish were placed at the end of the long arm. After a 1 min acclimation period, the sliding door was opened to allow exploration. During the initial 3‐day training period, zebrafish were conditioned to associate punishment with entry into the red arm and reward with entry into the green arm. On the final test day, fish were allowed to explore the maze freely without reward or punishment for 4 min. The time spent in each arm and transfer latency were recorded.

### 2.5 Evaluation of Cholinergic Function and Oxidative Stress Parameters

Following behavioral tests, zebrafish were euthanized using an ice‐water bath. Brain tissues were rapidly dissected, snap‐frozen in liquid nitrogen, and stored at −80°C until analysis. Brain tissues were homogenized on ice in ice‐cold physiological saline at a ratio of 1:9 (*w/v*). The homogenates were centrifuged at 12,000 rpm for 15 min at 4°C, and the supernatants were collected. Protein concentration was determined using the BCA method for normalization. The activities of AChE and ChAT, the levels of SOD and GSH, and the content of ACh and MDA were measured according to the manufacturers' instructions.

### 2.6 Gene Expression Analysis

To evaluate the neuroprotective effects of C3R, C3S, and C3X, mRNA expression levels of target genes were analyzed by quantitative real‐time PCR (qRT‐PCR). Target genes included those associated with cholinergic function (*ache* and *chata*), the APP protein family (*aplp2*), neurotrophic signaling (*bdnf* and *ntrk2b*), neuroinflammation (*il6, il1b, tnfa*, and *rela*), and the PI3K/Akt/GSK3β signaling pathway (*pik3r1*, *akt2*, and *gsk3ba*). Total RNA was extracted from zebrafish brain tissues using TRIzol reagent. RNA quality was confirmed (A260/A280 ≥ 1.8) before reverse transcription into cDNA according to the manufacturer's instructions. qPCR was performed in technical triplicate using the SYBR Green I method. Relative mRNA expression levels were calculated using the 2^−ΔΔ*Ct*^ method, with *actb1* (β‐actin) as the reference gene. Primer sequences are provided in Table S1.

### 2.7 Hematoxylin–Eosin (H&E) Staining and Nissl Staining

Histopathological analysis was performed to evaluate neuronal morphology and tissue damage. Following the experiment, zebrafish brains were fixed in 4% paraformaldehyde overnight. The tissues were dehydrated through a graded ethanol series, embedded in paraffin, and sectioned at 5 μm thickness. Sections were deparaffinized, rehydrated, and stained with H&E according to standard procedures to assess tissue architecture and pathological changes. Adjacent sections were stained with toluidine blue for Nissl staining to visualize Nissl bodies in neuronal cells. All stained sections were dehydrated, cleared, and mounted with neutral balsam. Images were captured at 200× magnification using a light microscope. Semi‐quantitative scoring of H&E staining and the percentage of positive staining area in Nissl staining were analyzed using Image‐Pro Plus 6.0 software (Media Cybernetics Inc., Rockville, MD, USA).

### 2.8 Transmission Electron Microscopy (TEM) Analysis

Adult zebrafish were euthanized by immersion in ice‐cold water (4°C). Brains were quickly isolated and fixed in 2.5% glutaraldehyde for 4 h at 25°C, followed by fixation at 4°C for 8 h. Samples were washed three times with cold (4°C) 0.1 M phosphate buffer (pH 7.4). Subsequently, tissues were dehydrated through graded ethanol and acetone series, embedded in resin, and cut into ultrathin sections (60 nm). Sections were stained and examined using a transmission electron microscope to observe ultrastructural changes in the zebrafish telencephalon.

### 2.9 Western Blot Analysis

Total proteins were extracted from adult zebrafish brain tissues, and protein concentration was determined using a BCA assay kit. Proteins were denatured, separated by 10% SDS‐polyacrylamide gel electrophoresis (SDS‐PAGE), and transferred onto PVDF membranes. Membranes were blocked with 5% non‐fat milk for 1 h and then incubated overnight at 4°C with primary antibodies (1:1000) against p‐Tau (Ser396), AChE, ChAT, p‐PI3K (Tyr458), PI3K p85, p‐Akt (Ser473), Akt, p‐GSK3β (Tyr216), GSK3β, and GAPDH. Band intensities of p‐PI3K (Tyr458), p‐Akt (Ser473), and p‐GSK3β (Tyr216) were normalized to their corresponding total proteins, whereas p‐Tau (Ser396), AChE, and ChAT were normalized to GAPDH. After washing with TBST, membranes were incubated with secondary antibodies (1:2000) for 1 h at room temperature. Following further washing, protein bands were visualized using an enhanced chemiluminescence (ECL) method and detected with an automated chemiluminescence imaging system (Tanon 5200). Band intensities were quantified using ImageJ software (Fiji, NIH, USA), with GAPDH as the internal control.

### 2.10 Immunofluorescence Staining

Paraffin‐embedded brain sections were deparaffinized and rehydrated through a graded ethanol series. Antigen retrieval was performed, followed by washing with PBS. Sections were outlined using a hydrophobic barrier pen and blocked with blocking buffer at room temperature for 30 min. Sections were then incubated overnight at 4°C with primary antibodies against AChE and ChAT. After washing with PBS, sections were incubated with fluorescently labeled secondary antibodies for 50 min at room temperature in the dark. Subsequently, nuclei were counterstained with DAPI. Sections were mounted with an anti‐fade mounting medium, and fluorescence images were obtained using a fluorescence microscope.

### 2.11 Molecular Docking

Comparative molecular docking was performed to explore the potential interactions of C3R, C3S, and C3X with human PI3Kα (PDB ID: 4JPS) and AKT1 (PDB ID: 3CQU) using AutoDock Vina 1.2.5. Detailed procedures for protein and ligand preparation and the docking grid parameters are provided in Method S1.

### 2.12 Statistical Analysis

All data are presented as mean ± SEM. Sample sizes and the numbers of independent biological samples are specified in the corresponding figure legends. Statistical analyses were performed using GraphPad Prism 9.4 (GraphPad Software, San Diego, CA, USA). Differences among groups were analyzed by one‐way analysis of variance (ANOVA) followed by Tukey's post hoc test for multiple comparisons. A value of *p* < 0.05 was considered statistically significant.

## 3 Results

### 3.1 Cyanidin Glycosides Ameliorate Learning and Memory Deficits in AlCl_3_ ‐Induced Zebrafish

The locomotor trajectories shown in Figure 2A illustrate the swimming patterns and object preference of zebrafish in each group. In the NOR test, the tank was virtually divided into familiar object (FO; red square) and novel object (NO; blue square) zones to assess object recognition behavior. Compared with the control group, AlCl_3_‐treated zebrafish showed a significant preference for the FO over the NO (*p* < 0.01, Figure 2C) and spent less time exploring the NO (*p* < 0.01, Figure 2B), indicating impaired learning and memory function. In contrast, treatment with C3R (*p* < 0.05), C3S (*p* < 0.05), C3X (*p* < 0.05), and Dpz (*p* < 0.01) significantly increased the time spent exploring the NO compared with the AlCl_3_ model group, thereby restoring preference for the NO over the FO (Figure 2B,C). Importantly, no significant differences in total distance traveled or average velocity were observed between treatment and model groups (Figure 2D,E), suggesting that the observed improvements in cognitive performance were not attributable to alterations in general locomotor activity.

**FIGURE 2:**
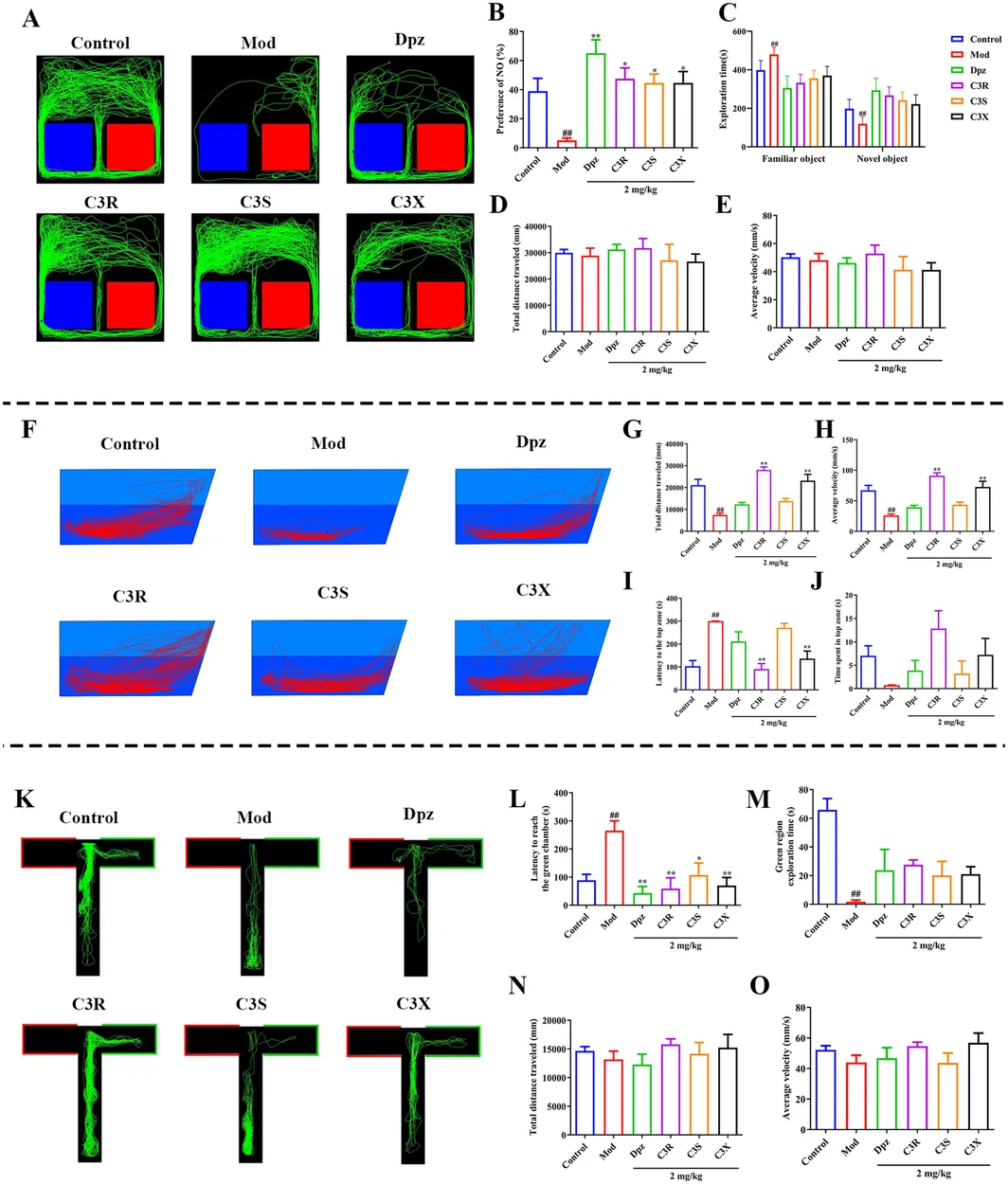
Effects of cyanidin glycosides on learning, memory, and anxiety‐like behavior in AlCl_3_‐induced zebrafish. (A) Representative locomotor trajectories in the NOR test. (B) Preference for the novel object (NO, %). (C) Exploration time of zebrafish in the familiar object (FO) and NO zones. (D) The total distance traveled by zebrafish in each group. (E) The average velocity of zebrafish in each group. (F) Representative locomotor trajectories in the NTT. (G) Total distance traveled. (H) Average velocity. (I) Latency to enter the top zone. (J) Time spent in the top zone. (K) Representative locomotor trajectories in the T‐maze test. (L) Latency to enter the green chamber. (M) Time spent in the green chamber. (N) Total distance traveled. (O) Average velocity. *n* = 6 per group. ^##^ *p* < 0.01 versus control group, *p < 0.05 and **p < 0.01 versus model group.

### 3.2 Cyanidin Glycosides Alleviate Anxiety‐Like Behavior in AlCl_3_ ‐Induced Zebrafish

Representative locomotor trajectories in the novel tank test are shown in Figure 2F. Compared with the control group, AlCl_3_‐treated zebrafish exhibited significantly reduced total distance traveled, average velocity, and frequency of entry into the top zone (*p* < 0.01, Figure 2F–H), indicating increased anxiety‐like behavior. In addition, AlCl_3_ exposure significantly prolonged the latency to enter the top zone and increased the preference for the bottom zone (*p* < 0.01, Figure 2I). Compared with the AlCl_3_ model group, treatment with C3R and C3X significantly shortened the latency to enter the top zone (*p* < 0.05, Figure 2J) and enhanced exploratory activity in the upper region. These results indicate that some cyanidin glycosides, particularly C3R and C3X, attenuated AlCl_3_‐induced anxiety‐like behavior in zebrafish. Although locomotor parameters were reduced in the model group, the combined changes in vertical exploration behavior (latency and time in the top zone) indicate an anxiety‐like phenotype rather than solely impaired motor function.

### 3.3 Cyanidin Glycosides Improve Learning and Memory Deficits in the T‐Maze Test

Representative tracking patterns in the T‐maze test are shown in Figure 2K. Compared with the control group, AlCl_3_‐treated zebrafish exhibited impaired learning and memory, as evidenced by prolonged latency to enter the green chamber and reduced preference for it (*p* < 0.01, Figure 2L,M). Consistently, locomotor trajectories revealed decreased exploration of the green chamber in the model group. In contrast, treatment with C3R (*p* < 0.01), C3S (*p* < 0.05), C3X (*p* < 0.01), and Dpz (*p* < 0.01) significantly reduced the latency to enter the green chamber (Figure 2L). Moreover, the cumulative time spent in the green chamber was significantly decreased in the model group (*p* < 0.01, Figure 2M), whereas it was markedly increased following treatment with cyanidin glycosides. These findings demonstrate that cyanidin glycosides improve spatial learning and memory performance in zebrafish. Furthermore, locomotor parameters, including total distance traveled and average velocity, were not significantly altered among groups (Figure 2N,O), indicating that the improved performance in the T‐maze was unlikely to be attributable to alterations in general locomotor activity.

### 3.4 Cyanidin Glycosides Attenuate Cholinergic Dysfunction and Oxidative Stress in AlCl_3_ ‐Induced Zebrafish

AlCl_3_ accumulation in the brain is known to induce cholinergic dysfunction and oxidative stress, contributing to neurodegeneration and cognitive impairment. To assess the protective effects of cyanidin glycosides, we measured cholinergic and oxidative stress‐related parameters in zebrafish. As shown in Figure 3A–C, AlCl_3_ exposure significantly increased AChE activity while decreasing ACh content and ChAT activity compared with the control group (*p* < 0.05). Treatment with C3R (*p* < 0.01), C3S (*p* < 0.05), C3X (*p* < 0.05), and Dpz (*p* < 0.05) partially reversed these alterations, consistent with the behavioral improvements. Furthermore, AlCl_3_ exposure significantly increased MDA levels and decreased SOD activity and GSH content (*p* < 0.01, Figure 3D–F). Treatment with C3R (*p* < 0.01), C3S (*p* < 0.05), C3X (*p* < 0.01), and Dpz (*p* < 0.01) significantly restored antioxidant capacity, as indicated by increased SOD and GSH levels and reduced MDA content. These results suggest that cyanidin glycosides effectively alleviate AlCl_3_‐induced cholinergic dysfunction and oxidative stress in zebrafish.

**FIGURE 3:**
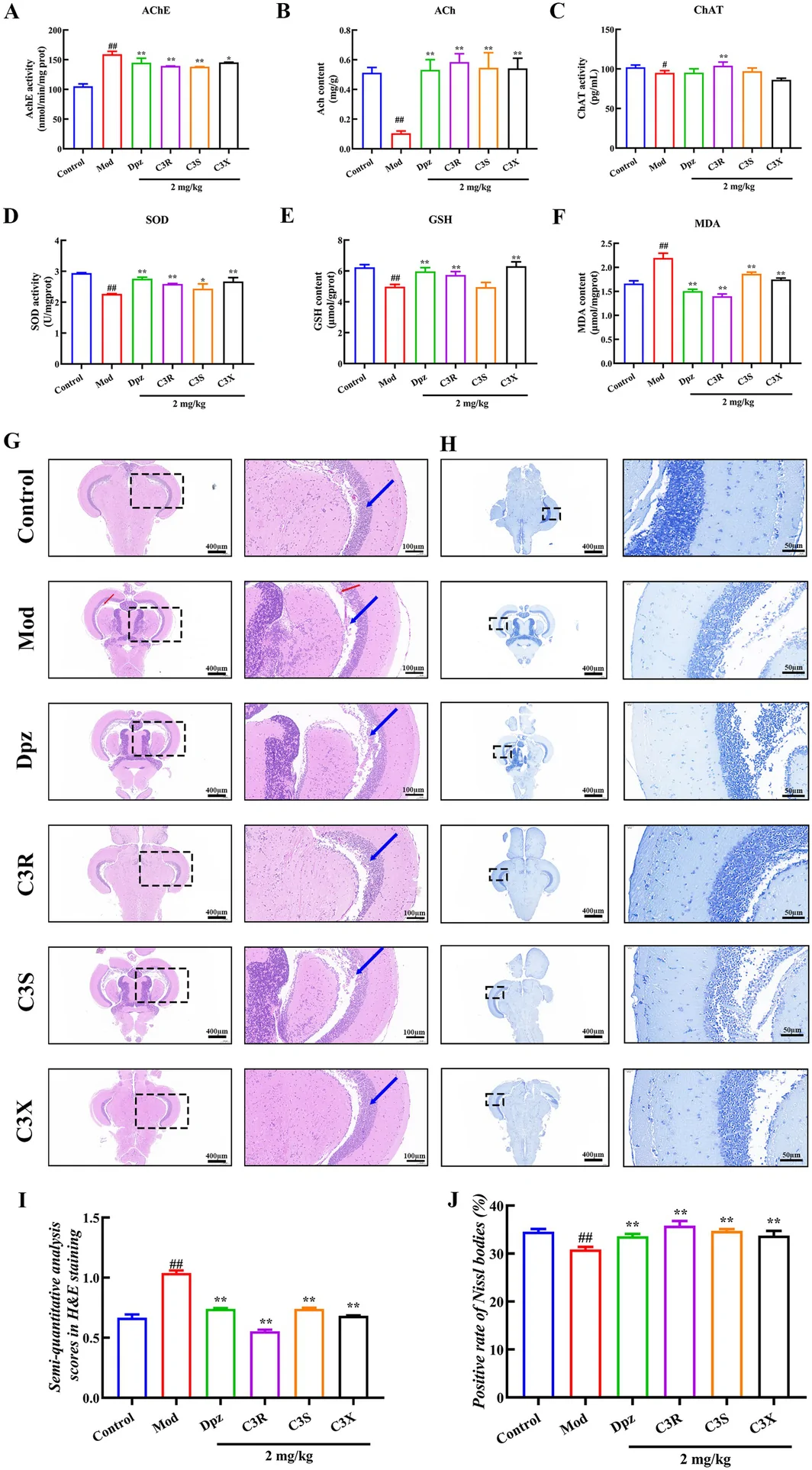
Effects of cyanidin glycosides on biochemical parameters and neuronal integrity in the brains of AlCl_3_‐induced zebrafish. (A) AChE activity. (B) ACh content. (C) ChAT activity. (D) SOD activity. (E) GSH content. (F) MDA level. (G) Representative H&E staining images of zebrafish brains. (H) Representative Nissl staining images. (I) Semi‐quantitative analysis of H&E staining. (J) Quantification of Nissl‐positive cells (%). *n* = 3 in each group, ^#^ *p* < 0.05 and ^##^ *p* < 0.01 versus control group, *p < 0.05 and **p < 0.01 versus model group.

### 3.5 Cyanidin Glycosides Attenuate Neuronal Damage in AlCl_3_ ‐Induced Zebrafish

H&E staining revealed that zebrafish in the control group exhibited a normal and intact brain structure, with densely arranged cells in the gray matter layer of the midbrain (Figure 3G). In contrast, the model group showed a loose cellular arrangement, reduced neuronal density, and signs of vascular congestion. Treatment with C3R, C3S, C3X, and Dpz markedly improved these histopathological alterations, as evidenced by increased cellular density and reduced tissue damage. Nissl staining further demonstrated that neurons in the control group were well‐organized with abundant Nissl bodies (Figure 3H). In contrast, the model group exhibited a significant reduction in Nissl bodies, along with disorganized and damaged neurons. Treatment with cyanidin glycosides and Dpz alleviated neuronal damage (*p* < 0.01, Figure 3I) and significantly increased the number of Nissl bodies (*p* < 0.01, Figure 3J). These results indicate that cyanidin glycosides effectively attenuate AlCl_3_‐induced neuronal damage in zebrafish.

### 3.6 Cyanidin Glycosides Protect Neuronal Ultrastructure in AlCl_3_ ‐Induced Zebrafish

Transmission electron microscopy (TEM) was used to examine neuronal cellular and organelle ultrastructure. In the control group, neurons displayed intact membrane boundaries and well‐organized intracellular structures, including mitochondria, endoplasmic reticulum, and Golgi apparatus (Figure 4A). In contrast, the model group showed marked ultrastructural damage, including disrupted membrane integrity, cytoplasmic vacuolization, cellular edema, mitochondrial swelling, and fewer identifiable organelles (Figure 4B). Treatment with Dpz, C3S, or C3X partially improved these changes, although mild edema, vacuolization, and reduced numbers of identifiable organelles were still observed (Figure 4C,E,F). Neurons in the C3R group showed more intact membrane boundaries, less cytoplasmic vacuolization, and better‐preserved mitochondria and other organelles (Figure 4D). These morphological observations indicate that cyanidin glycosides protect against AlCl_3_‐induced neuronal ultrastructural damage, with C3R showing the most evident preservation under the present experimental conditions.

**FIGURE 4:**
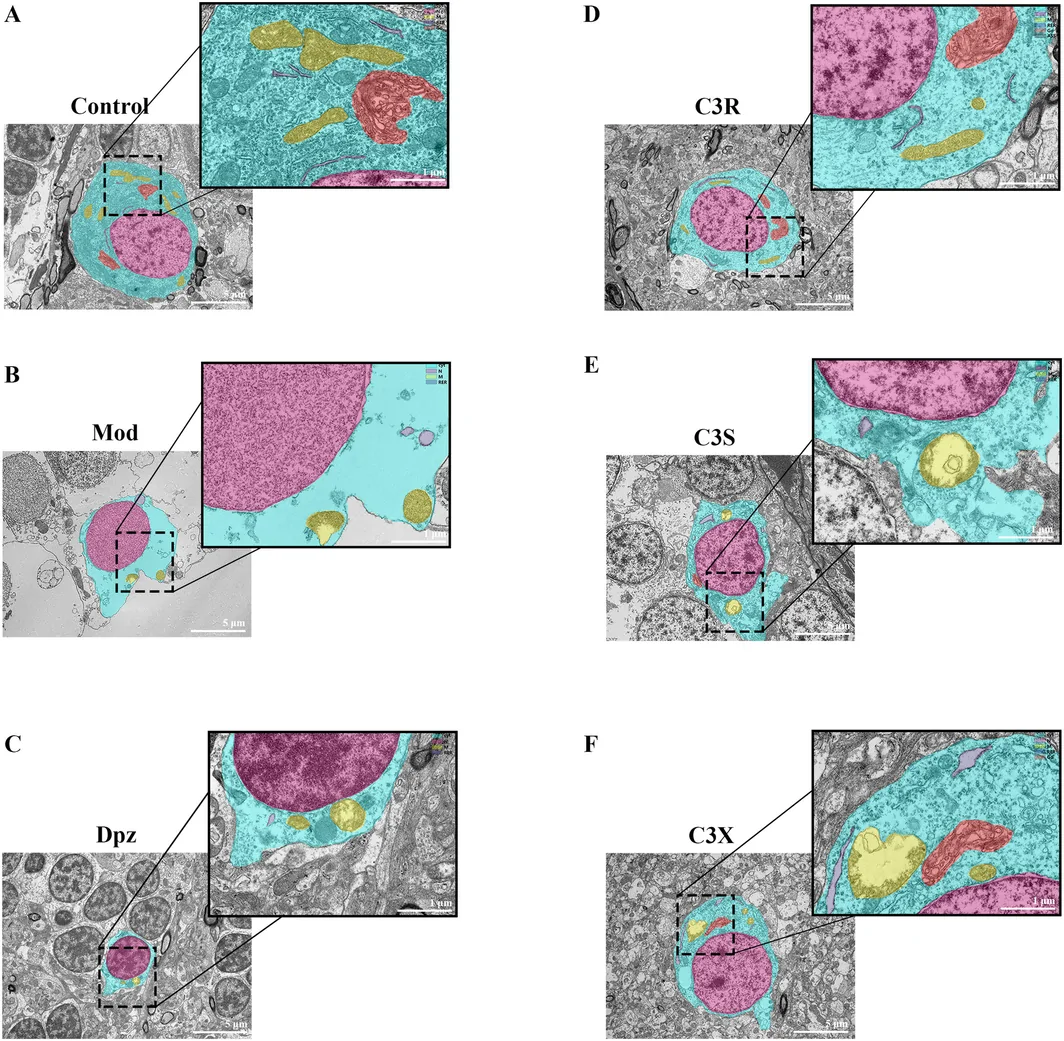
Ultrastructural changes in zebrafish brain neurons observed by TEM. Representative TEM images (×1500, scale bar = 5 μm) and enlarged views (×6000, scale bar = 1 μm). (A) Control group. (B) Model group. (C) Model + Dpz group. (D) Model + C3R group. (E) Model + C3S group. (F) Model + C3X group. Colored annotations indicate cytoplasm (green), nucleus (pink), mitochondria (yellow), Golgi apparatus (red), and endoplasmic reticulum (purple).

### 3.7 Cyanidin Glycosides Regulate mRNA Expression in AlCl_3_ ‐Induced Zebrafish

To evaluate the protective effects of C3R, C3S, and C3X against AlCl_3_‐induced damage, the expression of AD‐related genes was analyzed. Compared with the control group, AlCl_3_ exposure significantly upregulated *ache* and *aplp2* (Figure 5A,C), while downregulating *chata*, *bdnf*, and *ntrk2b* (Figure 5B,D,E). The expression levels of *il6*, *il1b*, *tnfa*, and *rela* were significantly increased (Figure 5F–I). In addition, *pik3r1* and *akt2* were downregulated, whereas *gsk3ba* was upregulated (Figure 5J–L). Treatment with C3R, C3S, C3X, and Dpz significantly reduced the expression of these pro‐inflammatory genes (*p* < 0.01). To further elucidate the underlying mechanisms, the expression of genes associated with the PI3K/Akt/GSK3β signaling pathway was examined (Figure 5J–L). The model group showed significant downregulation of *pik3r1* and *akt2* (*p* < 0.01), along with upregulation of *gsk3ba* (*p* < 0.01). Treatment with cyanidin glycosides and Dpz restored the expression levels of *pik3r1*, *akt2*, and *gsk3ba* toward normal levels (*p* < 0.01).

**FIGURE 5:**
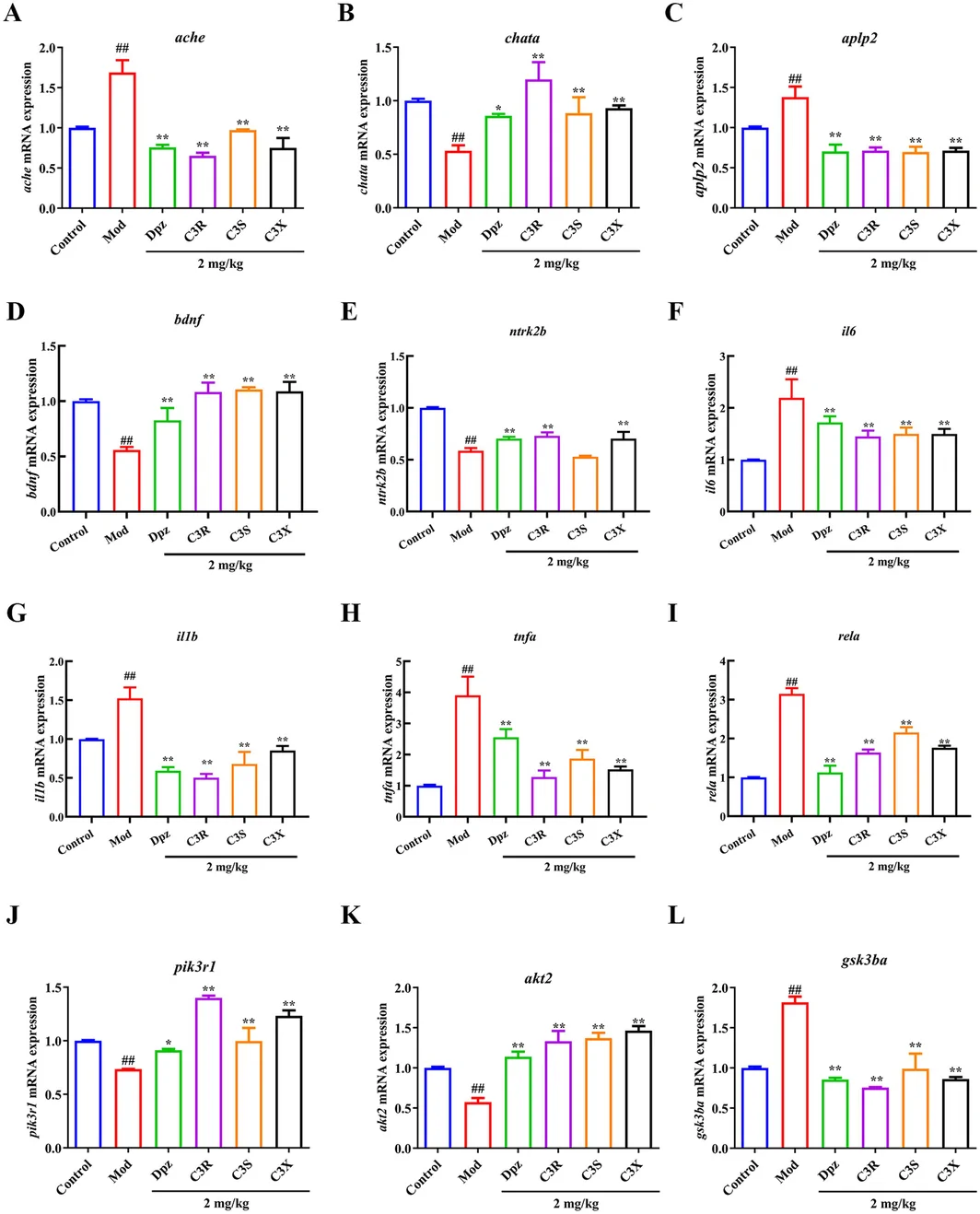
Effects of cyanidin glycosides on the relative mRNA expression of cholinergic, APP‐family‐related, neurotrophic, inflammatory, and PI3K/Akt/GSK3β pathway‐related genes in AlCl_3_‐induced zebrafish. (A) *ache*; (B) *chata*; (C) *aplp2*; (D) *bdnf*; (E) *ntrk2b*; (F) *il6*; (G) *il1b*; (H) *tnfa*; (I) *rela*; (J) *pik3r1*; (K) *akt2*; and (L) *gsk3ba*. *n* = 6 per group. ^##^ *p* < 0.01 versus control group, *p < 0.05 and **p < 0.01 versus model group.

### 3.8 Cyanidin Glycosides Modulate PI3K/Akt/GSK3β Signaling in the AlCl_3_ ‐Induced AD‐Like Zebrafish Model

To further investigate the underlying mechanism, protein expression levels of cholinergic markers and AD‐related proteins were analyzed by Western blot. As shown in Figure 6A,B, AlCl_3_ exposure induced cholinergic dysfunction, characterized by increased AChE expression and decreased ChAT expression (*p* < 0.01). Treatment with C3R, C3S, C3X, and Dpz significantly reversed these changes (*p* < 0.01). Moreover, AlCl_3_ exposure significantly increased p‐Tau (Ser396) expression (*p* < 0.01), whereas treatment with cyanidin glycosides and Dpz markedly reduced p‐Tau (Ser396) levels compared with the model group (*p* < 0.01). To assess the involvement of the PI3K/Akt/GSK3β pathway, the ratios of phosphorylated to total proteins were analyzed. In the model group, the p‐PI3K (Tyr458)/PI3K and p‐Akt (Ser473)/Akt ratios were significantly decreased, whereas the p‐GSK3β (Tyr216)/GSK3β ratio was significantly increased (*p* < 0.01), indicating enhanced activation‐associated phosphorylation of GSK3β. Treatment with C3R, C3X, and Dpz significantly reversed these alterations (*p* < 0.01). C3S significantly increased p‐PI3K (Tyr458) and decreased p‐GSK3β (Tyr216), but did not significantly restore p‐Akt (Ser473) in the primary comparison.

**FIGURE 6:**
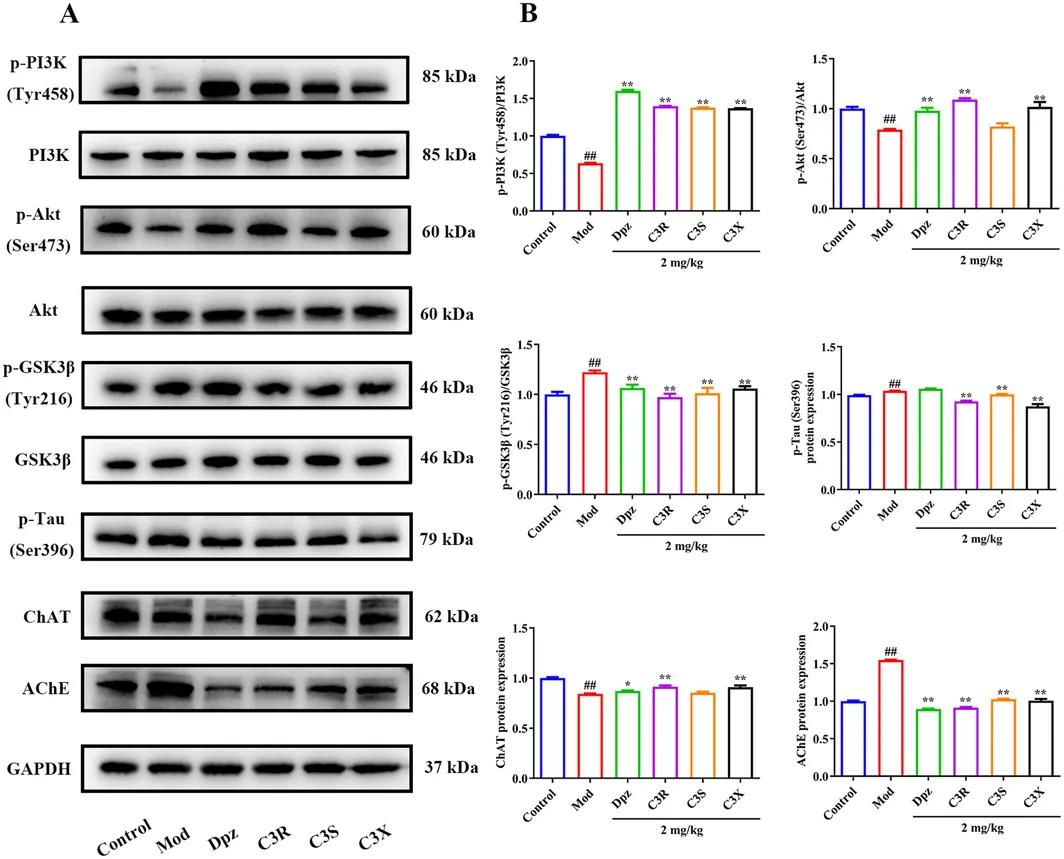
Effects of cyanidin glycosides on Tau phosphorylation and PI3K/Akt/GSK3β signaling. (A) Representative Western blot images of pathway‐related and cholinergic proteins. (B) Quantitative analysis of protein expression. *n* = 6 per group. ^##^ *p* < 0.01 versus control group, *p < 0.05 and **p < 0.01 versus model group.

To further validate pathway involvement, zebrafish were pretreated with the PI3K inhibitor LY294002 prior to cyanidin glycoside administration. As shown in Figure 7A,B, LY294002 markedly attenuated the increases in ChAT, p‐PI3K, and p‐Akt induced by C3R, C3S, and C3X, while increasing AChE, p‐GSK3β, and p‐Tau (Ser396) expression (*p* < 0.01). No significant changes were observed in total PI3K, Akt, or GSK3β levels among the groups. These results indicate that the effects of cyanidin glycosides are associated with modulation of PI3K/Akt signaling and attenuation of GSK3β Tyr216 and Tau Ser396 phosphorylation. To explore whether differences in the sugar moieties could contribute to the compound‐specific pathway responses, comparative molecular docking was performed against PI3Kα and AKT1. For PI3Kα, C3R showed the most favorable predicted docking score (−9.315 kcal/mol), followed by C3X (−8.567 kcal/mol) and C3S (−8.002 kcal/mol). C3R also showed the most favorable predicted score for AKT1 (−8.792 kcal/mol), compared with C3S (−7.956 kcal/mol) and C3X (−7.650 kcal/mol). The predicted binding poses further revealed distinct orientations and interaction patterns associated with the different sugar moieties (Figure S2). Thus, C3R exhibited the most favorable predicted molecular accommodation for both proteins examined. Nevertheless, these computational results do not establish direct binding or functional regulation of PI3Kα or AKT1.

**FIGURE 7:**
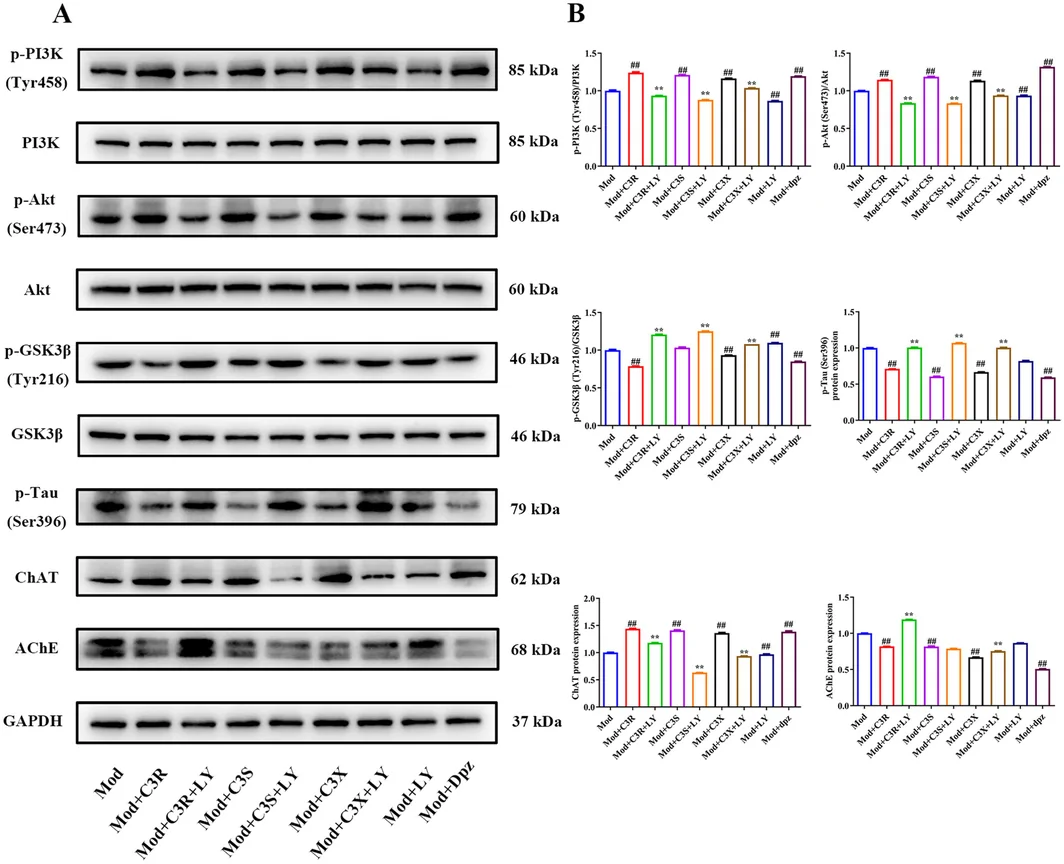
Inhibition of the PI3K/Akt/GSK3β pathway attenuates the protective effects of cyanidin glycosides in AlCl_3_‐induced zebrafish. (A) Representative Western blot images. (B) Quantitative analysis of protein expression. *n* = 6 per group.  ^##^ *p* < 0.01 versus model group, *p < 0.05 and **p < 0.01 versus model group.

### 3.9 Cyanidin Glycosides Restore Cholinergic Markers as Revealed by Immunofluorescence

To further validate the protective effects of cyanidin glycosides on the cholinergic system, immunofluorescence staining was performed to examine the expression of AChE and ChAT in zebrafish brain tissues. In the control group, AChE immunofluorescence signals were relatively weak, whereas ChAT exhibited strong positive staining, indicating intact cholinergic neurotransmission. In contrast, AlCl_3_ exposure markedly increased AChE fluorescence intensity and reduced ChAT expression, suggesting cholinergic dysfunction and synaptic impairment. Treatment with Dpz, C3R, C3S, and C3X significantly reversed these alterations. Compared with the model group, AChE fluorescence intensity was reduced, while ChAT expression was restored in all treatment groups (Figure 8). Among them, C3R exhibited relatively greater effects compared with the other cyanidin glycosides, with AChE levels approaching normal and substantial recovery of ChAT expression. Quantitative analysis further confirmed these observations. Compared with the model group, C3R significantly decreased AChE fluorescence intensity (*p* < 0.01) and increased ChAT expression (*p* < 0.01), whereas C3S and C3X also showed significant but less pronounced effects (*p* < 0.05). These results further support that cyanidin glycosides, particularly C3R, effectively restore cholinergic function and synaptic integrity in AlCl_3_‐induced zebrafish.

**FIGURE 8:**
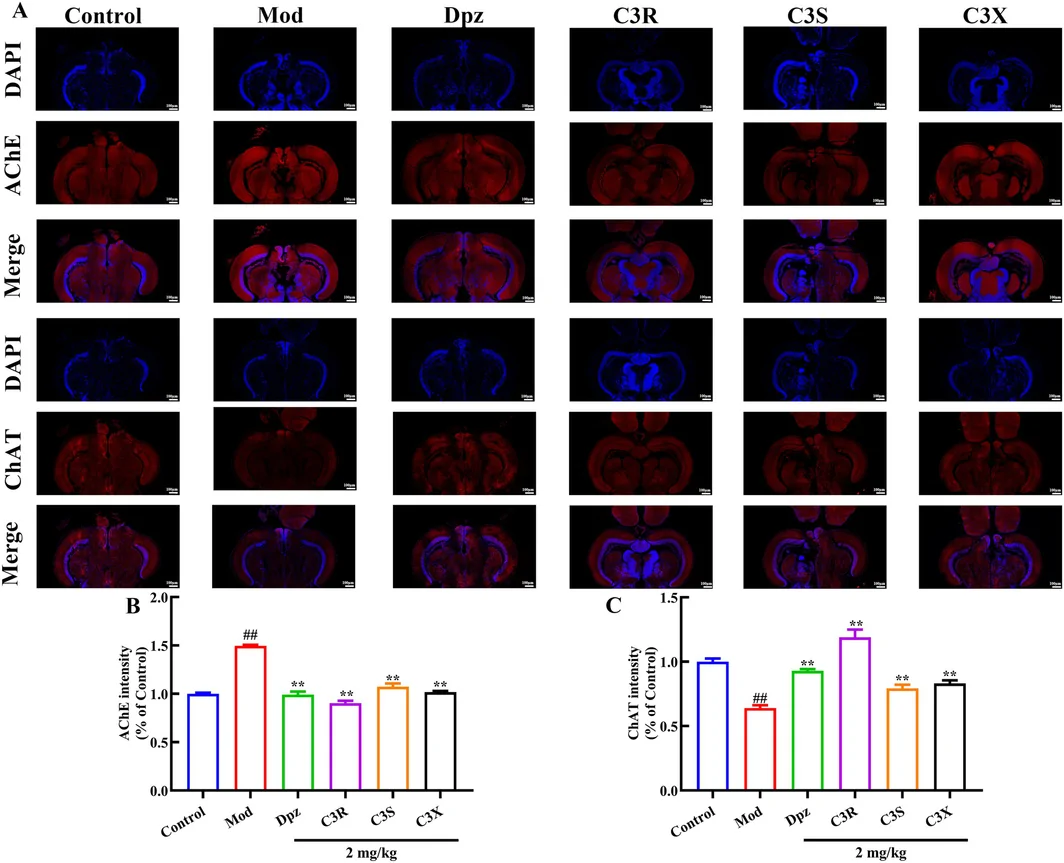
Immunofluorescence analysis of AChE and ChAT expression in zebrafish brains. (A) Representative immunofluorescence images. (B) Quantification of AChE fluorescence intensity. (C) Quantification of ChAT fluorescence intensity. Scale bar = 100 μm. *n* = 3 in each group, ^##^ *p* < 0.01 versus control; **p < 0.01 versus model.

## 4 Discussion

Despite extensive research, the pathogenic mechanisms of AD remain complex and incompletely understood, involving cholinergic dysfunction, Tau hyperphosphorylation, oxidative stress, and neuroinflammation (Nasb et al. 2024). In this context, dietary polyphenols, including anthocyanins, have attracted increasing attention because of their potential neuroprotective effects (Marino et al. 2022; Zhang and Yan 2023). Our previous comparative screening evaluated five cyanidin derivatives, including C3GG, C3R, C3A, C3S, and C3X, and identified C3R, C3S, and C3X as the most promising candidates for further investigation (Wu et al. 2025); however, their neuroprotective mechanisms remain unclear, including whether modulation of cholinergic function and AChE activity contributes to these effects. In the present study, we used an in vivo zebrafish model together with behavioral, biochemical, histological, and molecular analyses to comparatively evaluate the protective effects of three cyanidin glycosides (C3R, C3S, and C3X) against AD‐like pathology and explore their associated mechanisms.

The short‐term AlCl_3_‐induced zebrafish model used in this study captured selected AD‐like phenotypes, including increased Tau Ser396 phosphorylation, oxidative stress, cholinergic dysfunction, and inflammatory responses (Kashif et al. 2026; Nadiga et al. 2024). As a cholinotoxicant, AlCl_3_ disrupts cholinergic neurotransmission and promotes oxidative stress and neuroinflammation (Kashif et al. 2026; Paul and Firdous 2026). Consistent with previous reports (Gao et al. 2022; Zhang et al. 2024), the model exhibited cognitive deficits and anxiety‐like behaviors. Across the T‐maze, NOR, and NTT paradigms, cyanidin glycoside treatment improved spatial and recognition memory and reduced anxiety‐like behavior in AlCl_3_‐exposed zebrafish.

Histological and ultrastructural analyses further demonstrated that cyanidin glycosides protected neuronal integrity in AlCl_3_‐exposed zebrafish. Histological examination showed reduced neuronal damage and better preservation of Nissl bodies after treatment. TEM further revealed improved membrane integrity, less cytoplasmic vacuolization, and better‐preserved mitochondrial and organelle morphology, particularly in the C3R group. As TEM provides morphological evidence of cellular and organelle preservation, these observations cannot determine whether mitochondrial fission/fusion or caspase‐dependent apoptosis was directly affected. Further molecular studies are required to clarify these mechanisms.

In parallel with these structural changes, AlCl_3_ exposure induced pronounced biochemical alterations. Aluminum can cross the blood–brain barrier and accumulate in the hippocampus (Dooka et al. 2025), thereby promoting excessive reactive oxygen species (ROS) production, impairing antioxidant defenses, and activating inflammatory mediators, which may collectively contribute to cognitive dysfunction (Oyagbemi et al. 2025; Weng et al. 2020). Treatment with C3R, C3S, and C3X restored antioxidant capacity and reduced lipid peroxidation, supporting their protective effects against oxidative damage. Oxidative stress is also closely associated with cholinergic dysfunction, a hallmark of AD pathology. In the present study, AlCl_3_ exposure increased AChE activity and decreased ChAT expression, indicating impaired cholinergic neurotransmission. Cyanidin glycosides reversed these biochemical alterations, while immunofluorescence analysis showed reduced AChE and restored ChAT expression in zebrafish brain tissue. Dpz also improved oxidative stress and inflammatory indices in addition to restoring cholinergic markers. As an AChE inhibitor, donepezil reduces acetylcholine breakdown and enhances cholinergic signaling. Previous studies have shown that cholinesterase inhibitors can activate PI3K/Akt signaling through nicotinic acetylcholine receptors, particularly α7nAChR (Moreira et al. 2022; Takada‐Takatori et al. 2006). This pathway is involved in the regulation of GSK3β, antioxidant responses, neuroinflammation, and neuronal survival. Therefore, the broad effects of Dpz observed in the present study may result from downstream responses to enhanced cholinergic signaling rather than direct regulation of each measured target.

Most previous studies have evaluated individual cyanidin glycosides, whereas the present in vivo study further evaluates the neuroprotective effects of structurally distinct cyanidin glycosides and their association with PI3K/Akt/GSK3β signaling. Oxidative stress can impair PI3K/Akt signaling and promote aberrant GSK3β activation, thereby contributing to Tau hyperphosphorylation and neuronal dysfunction (Pan et al. 2024; Zheng et al. 2024). In line with this mechanism, AlCl_3_ exposure decreased the phosphorylation of PI3K p85 at Tyr458 and Akt at Ser473, while increasing the activation‐associated phosphorylation of GSK3β at Tyr216 and Tau phosphorylation at Ser396. C3R and C3X consistently reversed these alterations, whereas C3S increased PI3K Tyr458 phosphorylation and reduced GSK3β Tyr216 and Tau Ser396 phosphorylation without significantly restoring Akt Ser473 phosphorylation, indicating a partial pathway response. GSK3β is differentially regulated by site‐specific phosphorylation: Ser9 phosphorylation is inhibitory, whereas Tyr216 phosphorylation is associated with increased kinase activity (Krishnankutty et al. 2017). Because Akt canonically regulates GSK3β through Ser9 rather than Tyr216, and GSK3β kinase activity was not directly measured, the observed changes should be interpreted as coordinated pathway‐associated regulation rather than direct Akt‐mediated phosphorylation of GSK3β at Tyr216. The attenuation of these protective effects by LY294002 further supports the functional involvement of PI3K/Akt signaling. Since this pathway also integrates insulin and other metabolic signals, whether systemic metabolic changes contributed to the pathway response remains to be clarified, as glucose and related metabolic parameters were not examined in the present study.

To explore whether structural differences might contribute to these compound‐specific pathway responses, comparative molecular docking was performed against PI3Kα and AKT1. C3R showed the most favorable predicted docking scores for both proteins. Structurally, the relatively flexible α‐L‐rhamnosyl‐(1 → 6)‐β‐D‐glucose unit of C3R and the 6‐deoxy methyl group of rhamnose may permit conformational accommodation involving both polar and hydrophobic contacts. By comparison, the β‐(1 → 2)‐linked diglucoside moiety of C3S and the single xyloside moiety of C3X confer different steric features and predicted interaction patterns. This interpretation is consistent with our previous AChE‐focused findings indicating that glycosylation patterns and linkage flexibility influence the target‐specific interactions of cyanidin derivatives (Wu et al. 2025). Nevertheless, molecular docking provides a plausible structural hypothesis rather than evidence of direct binding or functional activation and cannot fully explain the stronger overall biological efficacy of C3R.

From a food science perspective, the present study identifies C3R as the most consistently effective of the three cyanidin glycosides under the same dose conditions, supporting its further investigation. The short‐term AlCl_3_‐induced zebrafish model provides a practical platform for rapid in vivo screening of neuroprotective compounds. However, it does not reproduce the chronic and age‐dependent development of human AD. The possible contribution of general antioxidant or aluminum‐interacting effects also cannot be excluded. Although gavage provides controlled oral administration, it does not fully reflect long‐term dietary intake within a complex food matrix. Species‐specific differences also limit the direct extrapolation of zebrafish pharmacokinetics to mammals (Poon et al. 2017). The gastrointestinal stability, bioavailability, metabolism, and brain exposure of these compounds remain to be determined. In addition, the LY294002 experiments support the involvement of PI3K/Akt/GSK3β signaling but do not identify the direct molecular targets of these compounds. The persistence of the behavioral improvements after compound withdrawal was not evaluated and remains to be determined. Future studies will include longer treatment periods and post‐treatment follow‐up. Mammalian AD models, including APP/PS1 mice, will also be used to evaluate pharmacokinetic properties, BBB penetration, and direct targets.

## 5 Conclusions

In summary, the three cyanidin glycosides protected against AD‐like pathology in zebrafish, with C3R showing the strongest overall efficacy. The findings provide preliminary in vivo evidence that their neuroprotective effects are associated with improvements in oxidative stress and cholinergic function, as well as modulation of PI3K/Akt/GSK3β signaling. Further mammalian pharmacokinetic and efficacy studies are needed before the dietary or functional food potential of C3R can be determined (Figure 9).

**FIGURE 9:**
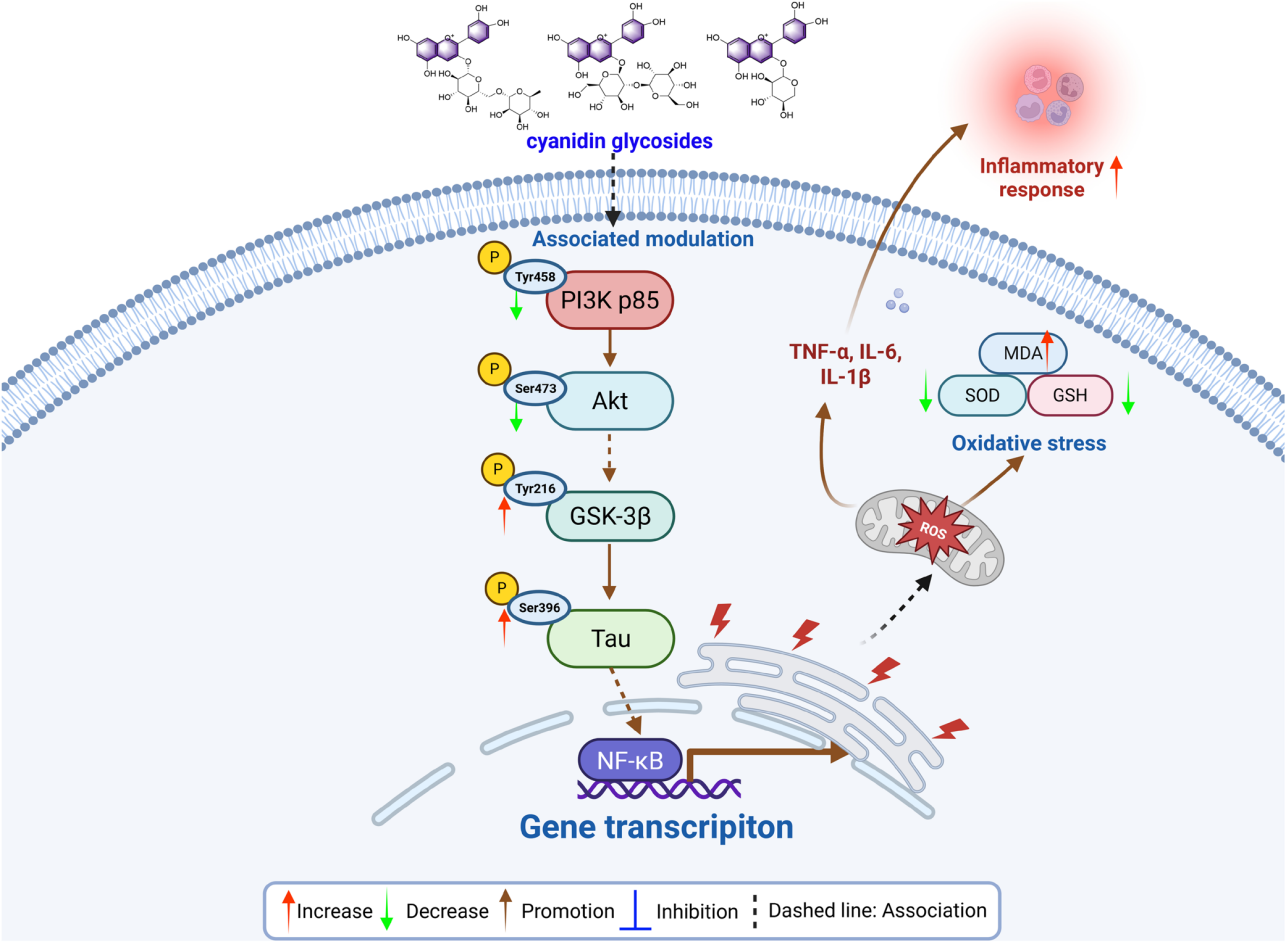
Schematic illustration of the proposed pathway‐associated neuroprotective effects of cyanidin glycosides against AlCl_3_‐induced AD‐like pathology in zebrafish. Red and green arrows indicate the changes induced by AlCl_3_ exposure, which were generally reversed by cyanidin glycoside treatment.

## Author Contributions

**Yun Wu:** conceptualization, writing – original draft. **Fangfang Tie:** writing – review and editing, data curation. **Yidan Gao:** methodology. **Jinze Li:** project administration. **Ruinan Wang:** data curation, validation. **Na Hu:** investigation. **Qi Dong:** software. **Honglun Wang:** resources, formal analysis, writing – review and editing.

## Ethics Statement

All animal experiments were conducted in accordance with the guidelines for the care and use of laboratory animals and were approved by the Animal Ethics Committee of the Northwest Institute of Plateau Biology, Chinese Academy of Sciences (Approval No. 2025‐37).

## Conflicts of Interest

The authors declare no conflicts of interest.

## Supporting information


**Figure S1:** Preliminary dose–response screening of Dpz and cyanidin glycosides in AlCl_3_‐exposed adult zebrafish.
**Figure S2:** Comparative molecular docking of C3R, C3S, and C3X with human PI3Kα and AKT1.
**Table S1:** Primer sequences used in the present study.
**Method S1:** Molecular docking.

## Data Availability

All data associated with this study are available from the corresponding author upon reasonable request.
