# Supplementary material for: Neuroprotective Effects of Food‐Derived Cyanidin Glycosides in an Alzheimer's Disease‐Like Zebrafish Model Through Modulation of PI3K/Akt/GSK3β Signaling
Source: Food Sci Nutr. 2026 Oct 15;14(10):e72486. doi: 10.1002/fsn3.72486 (PMC13671879; doi:10.1002/fsn3.72486)
Supplement: Supplementary file 1 [file FSN3-14-e72486-s001.docx]

**Neuroprotective effects of food-derived cyanidin glycosides in an Alzheimer’s disease-like zebrafish model through modulation of PI3K/Akt/GSK3β signaling**

Yun Wu ^a,b^, Fangfang Tie ^a^, Yidan Gao ^a^, Jinze Li ^a^, Ruinan Wang ^a^, Na Hu ^a^, Qi Dong ^a^, Honglun Wang ^a*^

**Supplementary materials**

**Supplementary Method S1. Molecular docking**

The crystal structures of human PI3Kα (PDB ID: 4JPS) and AKT1 (PDB ID: 3CQU) were retrieved from the RCSB Protein Data Bank and inspected using PyMOL 2.3.0. The three-dimensional structures of C3R, C3S, and C3X were obtained from the PubChem database and energy-minimized using the MMFF94 force field in Open Babel 3.1.1. Hydrogen atoms were added to the receptor structures, and hydrogen atoms and rotatable bonds were assigned to the ligands using AutoDockTools 1.5.6. The prepared receptor and ligand structures were subsequently converted to PDBQT format.

Semi-flexible molecular docking, in which the receptor was treated as rigid and the ligand as flexible, was performed using AutoDock Vina 1.2.5 with an exhaustiveness value of 25. For PI3Kα, the docking grid was centered at x = −11, y = −4, and z = 16, with dimensions of 33 × 20 × 27 Å. For AKT1, the grid was centered at x = 6, y = 0, and z = 19, with dimensions of 26 × 26 × 24 Å. The predicted docking poses and molecular interactions were visualized using PyMOL 2.3.0 and Discovery Studio 2019.

Table S1. Primer sequences used in the present study.

| **Gene symbol** | **Primer sequence (5′–3′)** |
| --- | --- |
| *actb1* | Forward: CGAGCAGGAGATGGGAACC |
|  | Reverse: CAACGGAAACGCTCATTGC |
| *ache* | Forward: CATACGCACAATACGCTGCC |
|  | Reverse: TACACAGCACCATGCGAGTT |
| *chata* | Forward: ATACGACCTACGCAACCAGC |
|  | Reverse: CGTAGCCATTTGGGACGACT |
| *aplp2* | Forward: AGCAGCTGGTGGAGACTCAT |
|  | Reverse: TAGTGGCGGATAGTGTGCTG |
| *bdnf* | Forward: CGAGGAATAGACAAGCGGCA |
|  | Reverse: ATCCGTATAAACCGCCAGCC |
| *ntrk2b* | Forward: TCACCTATGGCAAGCAACCC |
|  | Reverse: CTTTGGGGCAAGTACGAGGT |
| *il6* | Forward: ATGCCATCCGCTCAGAAAACAG |
|  | Reverse: CACATCCTGAACTTCGTCTCCA |
| *il1b* | Forward: TCGCCCAGTGCTCCGGCTAC |
|  | Reverse: GCAGCTGGTCGTATCCGTTTGG |
| *tnfa* | Forward: AAGGAGAGTTGCCTTTACCG |
|  | Reverse: ATTGCCCTGGGTCTTATGG |
| *rela* | Forward: CGCAAGAGAACTGAAGGAA |
|  | Reverse: AGAAAAAGGAGGTGGGTGG |
| *pik3r1* | Forward: GAGATTTTCTCGGCCCTGGCT |
|  | Reverse: ACTCTTCCCATCTGTGTGAGGC |
| *akt2* | Forward: CGGAGGTCCTGAAGATGC |
|  | Reverse: CTTGAAGGGTGGAACGAG |
| *gsk3ba* | Forward: GCCACAGAAACCAAGAGAGC |
|  | Reverse: GAATGCAAACGGAAGTGGAT |

**Figure S1. Preliminary dose-response screening of Dpz and cyanidin glycosides in AlCl_3_-exposed adult zebrafish.** (A) AChE activity. (B) ChAT activity. (C) SOD activity. (D) GSH level. (E) MDA level. The data are presented as the mean ± SEM (*n* = 3 in each group), ^#^*P* < 0.05 and ^##^*P* < 0.01 vs control group, ^*^*P* < 0.05 and ^**^*P* < 0.01 vs model group.

**Figure S2. Comparative molecular docking of C3R, C3S, and C3X with human PI3Kα and AKT1.** (A–C) Predicted binding modes of C3R, C3S, and C3X with PI3Kα, with docking scores of −9.315, −8.002, and −8.567 kcal/mol, respectively. (D–F) Predicted binding modes of C3R, C3S, and C3X with AKT1, with docking scores of −8.792, −7.956, and −7.650 kcal/mol, respectively. Each panel shows the predicted ligand position within the protein pocket together with two-dimensional and three-dimensional interaction maps indicating the predicted hydrogen-bonding, π-related, and hydrophobic contacts with surrounding amino acid residues.
